# COVIDMED – An early pandemic randomized clinical trial of losartan treatment for hospitalized COVID-19 patients

**DOI:** 10.1016/j.conctc.2022.100968

**Published:** 2022-07-20

**Authors:** Daniel Freilich, Jennifer Victory, Paul Jenkins, Anne Gadomski

**Affiliations:** a Bassett Medical Center, 1 Atwell Rd, Cooperstown, NY, 13326, USA; b Bassett Research Institute, 1 Atwell Rd, Cooperstown, NY, 13326, USA

**Keywords:** Losartan, Angiotensin converting enzyme inhibitor (ACEi), Angiotensin II receptor Blocker (ARB), COVID-19, SARS-CoV-2, Randomized clinical trial (RCT), COSS, COVID-19 Ordinal Severity Score

## Abstract

**Objectives:**

To assess the efficacy and safety of losartan for COVID-19 patients.

**Methods:**

COVIDMED was a double-blinded, placebo-controlled platform RCT. Enrollees were randomized to standard care plus hydroxychloroquine, lopinavir/ritonavir, losartan, or placebo. Hydroxychloroquine and lopinavir/ritonavir arms were discontinued early. We report losartan data vs. combined (lopinavir-ritonavir and placebo) and prespecified placebo-only controls. The primary endpoint was the mean COVID-19 Ordinal Severity Score (COSS) slope of change. Slow enrollment prompted early termination.

**Results:**

Fourteen patients were included in our final analysis (losartan [N = 9] vs. control [N = 5] [lopinavir/ritonavir [N = 2], placebo [N = 3]]). Most baseline parameters were balanced. Losartan treatment was not associated with a difference in mean COSS slope of change vs. combined (p = 0.4) or placebo-only control (p = 0.05) (trend favoring placebo). 60-day mortality and overall AE/SAE rates were insignificantly higher with losartan.

**Conclusion:**

In this small RCT in hospitalized COVID-19 patients, losartan did not improve outcome and was associated with adverse safety signals.

## 1 Introduction

Angiotensin II receptor blockers (ARB) may benefit COVID-19 patients secondary to pneumocyte ACE-2 receptor and SARS-CoV-2 entry inhibition, vasodilation/vasoconstriction alteration, and anti-inflammatory effects [1]. Although animal studies suggest potential for benefit, safety concerns include potential for increased ACE-2 receptor expression and adverse events [[2], [3], [4]]. Retrospective/observational exposure-nonexposure and continuation-discontinuation trials [[5], [6], [7], [8], [9], [10]], open-label interventional trials [[11], [12], [13], [14], [15]], and a meta-analysis [16] showed potential benefit. Two blinded RCTs, however, showed no benefit yet adverse safety signals [17,18]. COVIDMED assessed the ARB losartan in hospitalized COVID-19 patients.

## 2 Materials and methods

COVIDMED (NCT04340557), a parallel-design blinded platform RCT, was approved by the IRBs of Bassett Medical Center (Cooperstown, NY [April 3, 2020] [#1581969]), and two other participating sites (ClinicalTrials.gov - NCT04328012). Our objective was to compare effects of hydroxychloroquine, lopinavir/ritonavir, or losartan, vs. placebo (all investigational unlabeled uses) on a COVID-19 Ordinal Severity Score (COSS) – 1) Death; 2) Hospitalized-on mechanical ventilation/ECMO; 3) Hospitalized-on NIV; 4) Hospitalized-requiring oxygen; 5) Hospitalized-not requiring oxygen; 6) Not hospitalized-with limitations; 7) Not hospitalized-without limitations.

Post-consent, patients were allocated in a 2:2:2:1 ratio in blocks to one of the above four treatment groups using a statistician/computer-generated randomization schedule (without stratification). Allocation concealment was ensured by having only the enrollment research nurse be unblinded and maintaining allocation confidentiality. All groups received standard care and were followed for 60 days.

Hydroxychloroquine and lopinavir/ritonavir enrollment were halted after RCTs showed no benefit [19,20]; a 2:1 losartan:placebo allocation schedule was used thereafter. We report trial design/results for losartan vs. combined control (including 2 lopinavir/ritonavir patients) and placebo-only groups (per our prespecified statistical analysis plan [SAP]). Low enrollment prompted study termination May 27, 2021.

Key inclusion criteria were: hospitalized; ≥18 years-old; laboratory-confirmed SARS-CoV-2; and randomization within 72 h. Key losartan group exclusion criteria included: taking ACEi/ARB; hypotension; hyperkalemia; renal dysfunction/volume depletion. There were no placebo group exclusions. After hydroxychloroquine/lopinavir-ritonavir enrollment ceased, inclusion/exclusion criteria were the same for losartan and placebo.

Study drug (losartan or TicTacs placebo [lopinavir-ritonavir in 2 patients]) was inserted into blank capsules by a pharmacist. Dosing was initially twice daily to mask varying regimens. Losartan and lopinavir/ritonavir doses were 25 mg and 400/100 mg, respectively. After hydroxychloroquine/lopinavir/ritonavir enrollment ceased, losartan and placebo dosing were daily for 5–14 days.

An arbitrary sample size of 4000 was chosen at the pandemic onset, with interim analysis optimization upon enrollment of 114 treatment and 57 placebos groups subjects, however, the study was terminated beforehand.

AEs were classified in accordance with NCICTC for Adverse Events, version 4.0. COVIDMED was carried out in accordance with principles of protection of humans participating in research, including the Declaration of Helsinki, and consent from all participants.

### 2.1 Statistical analysis

Mean slopes of change in COSS over time served as the primary endpoint for each subject. Continuous and categorical secondary endpoints were compared using Student's t-test and Fisher's exact test. Small N prompted omitting adjusted and subgroup analyses. Primary outcome missingness was addressed with last-observation-carried-forward. SAP modifications included Student's t-test use for primary analysis, losartan vs. combined control comparison, and per protocol reporting.

## 3 Results

### 3.1 Screening/enrollment

Of 448 screened patients, 15 were enrolled (3.3%), 9 receiving losartan and 6 receiving control (placebo = 4, lopinavir/ritonavir = 2); 1 placebo patient who withdrew after enrollment but prior to study drug was excluded (yielding 5 control patients [placebo = 3, lopinavir/ritonavir = 2]). Reasons for non-inclusion: patient declined (12.0%), taking ACEi/ARB (26.8%), outside enrollment window (20.1%), hypotension (1.6%), hyperkalemia (2.1%), renal disease (4.2%), altered mental status (11.5%), unrelated hospitalization (10.4%), withdrawal post-consent/pre-randomization (0.2%), and other (11.1%) (Supplement 1).

### 3.2 Baseline parameters/demographics

Baseline data were reasonably balanced (Table 1).

**Table 1:** Baseline parameters/demographics. Losartan is compared with combined control (lopinavir/ritonavir and placebo) and placebo only control. Statistically significant and ‘trend’ comparisons are in bold.

	**Treatment**	**Combined control**	**p**	**95% CI**	**Placebo only control**	**p**	**95% CI**
	**Losartan**	**Lopinavir/ritonavir and placebo**			**Placebo**		
N	9	5	NA	NA	3	NA	NA
Age (mean)	63.7	61.8	0.8	−17.0153 to 13.2153	56.3	0.4	−27.4633 to 12.6633
Male (%)	66.7	60.0	0.9	−0.80986 to 0.94319	66.7	NA	NA
Enrollment spring 2020	11.1	60	0.1	−1.0732 to 0.0955	33.3	0.4	−0.7557 to 0.3112
Enrollment fall 2020 - winter 2021	77.8	20	0.2	−0.2486 to 1.4042	33.3	0.4	−0.6224 to 1.5113
Enrollment spring 2021	11.1	20	0.7	−0.50209 to 0.32431	33.3	0.4	−0.7557 to 0.3112
Caucasian ethnicity (%)	100	100	NA	NA	100	NA	NA
COSS (mean)	3.6	4.0	0.5	−0.7192 to 1.5192	3.7	0.9	−1.2961 to 1.4961
Symptoms duration, days (mean)	9.3	7.4	0.3	−6.0157 to 2.315	6.7	0.3	−7.9688 to 2.8688
Comorbidites (targeted) rate (mean)	1.0	2.6	**0.02**	0.3255 to 2.8745	2.7	**0.046**	0.0392 to 3.3608
Immunocompromised (%)	22.2	60	0.3	−1.0311 to 0.2755	66.7	0.2	−1.1988 to 0.3099
Chronic heart disease (%)	22.2	80	0.1	−1.2935 to 0.1379	66.7	0.2	−1.1988 to 0.3099
Chronic lung disease (%)	33.3	60	0.5	−0.9823 to 0.449	66.7	0.5	−1.1768 to 0.5101
Chronic kidney disease (%)	0	0	NA	NA	0	NA	NA
Chronic liver disease (%)	0	0	NA	NA	0	NA	NA
Diabetes (%)	22.2	25	0.9	−0.48384 to 0.52828	33.3	0.7	−0.7644 to 0.5422
Extreme obesity (%)	0	50	**0.03**	−0.962 to −0.038	50	**0.03**	−0.962 to −0.038
Charlson Score (mean)	2.5	4.5	**0.08**	−0.3196 to 4.3196	3.7	0.3	−1.1366 to 3.5366
qSOFA (mean)	0.33	0.6	0.4	−0.3585 to 0.8985	0.3	1	−0.7680 to 0.7680
BMI (mean)	31.0	31.8	0.4	−4.2764 to 9.2764	35.1	0.2	−2.3076 to 10.5076
Creatinine (mean)	0.7	0.9	0.4	−0.2508 to 0.5708	0.7	0.9	−0.2127 to 0.2327
Chest x-ray opacities (%)	88.9	100	0.9	−1.1646 to 0.9423	100	0.9	−1.3621 to 1.1399
Pneumonia severity index (PSI) (mean)	54.9	59.3	0.5	−41.3071 to 50.1071	60	0.8	−40.4537 to 21.2537
Treatment days (mean)	9.6	13.3	0.3	−3.5545 to 10.8545	13	0.4	−5.1086 to 11.9086
Treatment with corticosteroids (%)	88.9	40	0.3	−0.7949 to 0.2172	60	0.7	−0.9706 to 1.415

### 3.3 Efficacy

96–97% of COSS timepoints were recorded. Mean slopes of change for COSS were 0.00365 for losartan (p = 0.5), 0.02091 for combined control (p = 0.07), and 0.05268 for placebo-only control (p = 0.002). Comparisons of slopes of change for COSS (primary outcome) revealed: losartan vs. combined control (p = 0.4), losartan vs. placebo-only control (p = 0.05) (trend favoring placebo), combined control vs. placebo-only control (p = 0.267) (Supplement 2).

60-day mortality (44.4 vs. 20%, p = 0.9), mean LOS (16.4 vs. 7.0 days, p = 0.2), and mean mechanical ventilation duration (8.5 vs. 0 days) were insignificantly higher with losartan (Table 2).

**Table 2:** Efficacy. Losartan is compared with combined control (lopinavir/ritonavir and placebo) and placebo only control. Statistically significant and ‘trend’ comparisons are in bold. Comparison of mean COSS slope of the change was the study's primary efficacy outcome measurement.

	**Treatment**	**Combined control**	**p**	**95% CI**	**Placebo only control**	**p**	**95% CI**
	**Losartan**	**Lopinavir/ritonavir and placebo**			**Placebo**		
COSS slope of the change	0.0037	0.0209	0.4		0.0527	**0.05**	
COSS change day 60	0.7	1.8	0.6	−2.8175 to 5.0175	3.3	0.2	−1.6690 to 6.8690
Mortality, 60 days (%)	44.4	20.0	0.9	−0.67123 to 0.76012	0	0.2	−0.3099 to 1.1988
First negative PCR, days (mean)	6.5	10.5	0.7	−12.3243 to 16.3243	3	NA	NA
Hospital LOS, days (mean)	16.4	7.0	0.2	−25.2922 to 6.4922	6.3	0.3	−28.8728 to 8.6728
Mechanical ventilation, days (mean)	8.5	0	NA	NA	0	NA	NA

### 3.4 Safety

The overall SAE rate was numerically higher with losartan vs. combined control (2.0 vs. 0.6%) and vs. the placebo-only control (2.0 vs. 0%). The overall AE rate trend was similar, being higher with losartan than combined control (3.9 vs. 1.0%) and vs. placebo-only control (3.9 vs. 0%). AKI AE and SAE rates were similar, but hypotension, hyperkalemia, and respiratory failure AE and SAE rates, were numerically higher with losartan than combined and placebo-only controls. No safety comparison was significantly different (Table 3).

**Table 3:** Safety. AE and SAE rates including relatedness. Losartan is compared with combined control (lopinavir/ritonavir and placebo) and placebo only control. Statistically significant and ‘trend’ comparisons are in bold. Comparison of SAE rate was the study's primary safety outcome measurement.

	**Losartan**					**Control (placebo or lopinavir/ritonavir)**					**p**	**95% CI**	**Total**	**Control (placebo)**					**95% CI**
	**Total**	**Definitely**	**Probably**	**Possibly**	**Not**	**Total**	**Definitely**	**Probably**	**Possibly**	**Not**				**Definitely**	**Probably**	**Possibly**	**Not**	**p**	
AEs/subject (mean)	3.9	0	0	1.0	2.9	1.0	0	0	1.0	0	0.3	−8.3414 to 2.5414	0	0	0	0	0	NA	NA
SAEs/subject (mean)	2.0	0	0	0.4	1.6	0.60	0	0	0.6	0	0.3	−4.3144 to 1.5144	0	0	0	0	0	NA	NA
AKI AEs/subject (mean)	0.22	0	0	0.11	0.11	0.20	0	0	0.2	0	0.95	−0.7560 to 0.7160	0	0	0	0	0	NA	NA
AKI SAEe/subject (mean)	0.11	0	0	0.00	0.11	0.20	0	0	0.2	0	0.5	−0.5160 to 0.9560	0	0	0	0	0	NA	NA
Hypotension AEs/subject (mean)	0.33	0	0	0.22	0.11	0	0	0	0	0	NA	NA	0	0	0	0	0	NA	NA
Hypotension SAEs/subject (mean)	0.22	0	0	0.11	0.11	0	0	0	0	0	NA	NA	0	0	0	0	0	NA	NA
Hyperkalemia AEs/subject (mean)	0.11	0	0	0.11	0	0	0	0	0	0	NA	NA	0	0	0	0	0	NA	NA
Hyperkalemia SAEs/subject (mean)	0.00	0	0	0	0	0	0	0	0	0	NA	NA	0	0	0	0	0	NA	NA
Respiratory failure/COVID-19 PNA AEs rate (mean)	0.67	0	0	0.33	0.33	0.20	0	0	0.2	0	0.2	−1.2420 to 0.3020	0	0	0	0	0	NA	NA
Respiratory failure/COVID-19 PNA SAEs rate (mean)	0.67	0	0	0.33	0.33	0.20	0	0	0.2	0	0.2	−1.2420 to 0.3020	0	0	0	0	0	NA	NA

## 4 Discussion

COVIDMED, the third blinded placebo-controlled RCT assessing an ARB in COVID-19, did not find significant group differences in COSS for hospitalized patients treated with losartan vs. control. An insignificant trend favoring control was found for our primary efficacy outcome, comparison of mean COSS slope of the change vs. placebo-only control (p = 0.05). Our primary safety outcome, overall SAE rate, was numerically but not significantly higher with losartan. Secondary outcomes also numerically favored placebo but no group comparisons were significantly different. We speculate that ARB class adverse effects may overcome theorized benefits making the benefit:risk ratio for these medications in COVID-19 null or negative.

Strengths of our study include: blinded RCT, minimal missingness, and baseline balance. Limitations include small N, early termination, low enrollment (reducing external validity), and SAP alterations.

## 5 Conclusion

Although COVIDMED was pilot-like in scope, its results add randomized, blinded, placebo-controlled data to the limited COVID-19 ACEi/ARB literature. Our results are similar to two larger blinded RCTs [17,18]. The totality of the data do not support empiric ACEi/ARB initiation in COVID-19 outside of RCTs.

## Source of funding

This work was supported by a Bassett Research Institute ED Thomas Grant (which included funds to purchase study drugs) and salary support from the Bassett Research Institute and Bassett Medical Center's Department of Internal Medicine. Funders had no role in the design, execution, data analysis, or manuscript preparation or publication of this study.

## Authors’ contributions

All authors contributed to refinement of and approved the manuscript.

## Authorship role

All authors collaborated in the writing/editing of the manuscript.

## Declaration of competing interest

The authors declare that they have no known competing financial interests or personal relationships that could have appeared to influence the work reported in this paper.

## Figures and Tables

**Figure:**
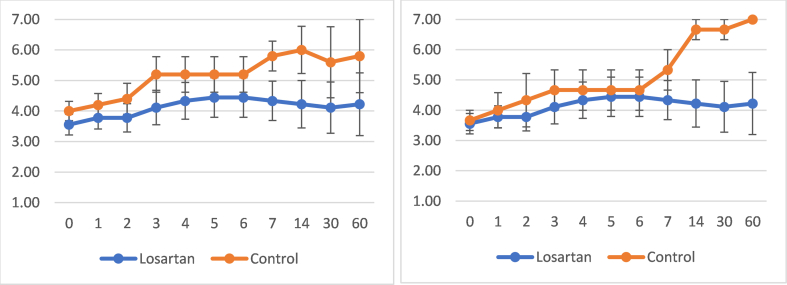
Multimedia component 3
