# Supplementary material for: COVIDMED – An early pandemic randomized clinical trial of losartan treatment for hospitalized COVID-19 patients
Source: Contemp Clin Trials Commun. 2022 Jul 20;29:100968. doi: 10.1016/j.conctc.2022.100968 (PMC9296371; doi:10.1016/j.conctc.2022.100968)
Supplement: Multimedia component 2 [file mmc2.doc]

**Figure 1. CONSORT 2010 Flow Diagram (**[**http://www.consort-statement.org/**](http://www.consort-statement.org/)**)**

**Allocation**

**Analysis**

**Follow-Up**

**Enrollment**

Assessed for eligibility (n=448)

Analysed (n=9)
♦ Excluded from analysis (n=0)

Lost to follow-up (n=0)

Discontinued intervention (withdrew prior to 60-day follow-up) (n=1)

Allocated to losartan (n=9)

♦ Received allocated intervention (n=9)

♦ Did not receive allocated intervention (n=0)

Randomized (n=15)

Excluded (n= 433)

♦  Not meeting inclusion criteria (n=332)

♦  Declined to participate (n=52)

♦  Other (n=48)

♦  Withdrew post-consent, pre-randomization (n=1)

Lost to follow-up (n=0)

Discontinued intervention (n=0)

Allocated to control (n=6)

(Placebo [n=4]), lopinavir/ritonavir [n=2])

♦ Received allocated intervention (n=5)

♦ Did not receive allocated intervention

(withdrew post-randomization, pre-study drug) (n=1)

Analysed (n=5)

(Placebo [n=3], lopinavir/ritonavir [n=2])
♦ Excluded from analysis (n=0)
